# Evaluation by dental professionals of an artificial intelligence-based application to measure alveolar bone loss

**DOI:** 10.1186/s12903-025-05677-0

**Published:** 2025-03-01

**Authors:** Sang Won Lee, Kateryna Huz, Kayla Gorelick, Jackie Li, Thomas Bina, Satoko Matsumura, Noah Yin, Nicholas Zhang, Yvonne Naa Ardua Anang, Sanam Sachadava, Helena I. Servin-DeMarrais, Donald J. McMahon, Helen H. Lu, Michael T. Yin, Sunil Wadhwa

**Affiliations:** 1 Department of Biomedical Engineering, Columbia University, New York, NY 10027 USA; 2 Division of Orthodontics, Columbia University College of Dental Medicine, New York, NY 10032 USA; 3 Division of Oral & Maxillofacial Radiology, Columbia University College of Dental Medicine, New York, NY 10032 USA; 4 Vagelos College of Physicians and Surgeons, Division of Infectious Diseases, Columbia University, New York, NY 10032 USA

**Keywords:** Alveolar crestal height, Periodontal disease, Artificial intelligence, Deep learning, Acceptability, Dental professionals

## Abstract

**Background:**

Several commercial programs incorporate artificial intelligence in diagnosis, but very few dental professionals have been surveyed regarding its acceptability and usability. Furthermore, few have explored how these advances might be incorporated into routine practice.

**Methods:**

Our team developed and implemented a deep learning (DL) model employing semantic segmentation neural networks and object detection networks to precisely identify alveolar bone crestal levels (ABCLs) and cemento-enamel junctions (CEJs) to measure change in alveolar crestal height (ACH). The model was trained and validated using a 550 bitewing radiograph dataset curated by an oral radiologist, setting a gold standard for ACH measurements. A twenty-question survey was created to compare the accuracy and efficiency of manual X-ray examination versus the application and to assess the acceptability and usability of the application.

**Results:**

In total, 56 different dental professionals classified severe (ACH > 5 mm) vs. non-severe (ACH ≤ 5 mm) periodontal bone loss on 35 calculable ACH measures. Dental professionals accurately identified between 35-87% of teeth with severe periodontal disease, whereas the artificial intelligence (AI) application achieved an 82–87% accuracy rate. Among the 65 participants who completed the acceptability and usability survey, more than half the participants (52%) were from an academic setting. Only 21% of participants reported that they already used automated or AI-based software in their practice to assist in reading of X-rays. The majority, 57%, stated that they only approximate when measuring bone levels and only 9% stated that they measure with a ruler. The survey indicated that 84% of participants agreed or strongly agreed with the AI application measurement of ACH. Furthermore, 56% of participants agreed that AI would be helpful in their professional setting.

**Conclusion:**

Overall, the study demonstrates that an AI application for detecting alveolar bone has high acceptability among dental professionals and may provide benefits in time saving and increased clinical accuracy.

**Supplementary Information:**

The online version contains supplementary material available at 10.1186/s12903-025-05677-0.

## Introduction

Significant advances in artificial intelligence (AI) have improved computer-aided diagnosis (CAD) for oral imaging [1–3], similar to advances made in other areas of medicine for radiographic images [1, 2, 4–12]. Deep learning (DL) models, encompassing tasks such as the identification of anatomical structures and the detection of pathological findings on radiographic images, have been widely and successfully utilized in medicine and in dentistry [1, 4–7]. The prevailing method for measuring alveolar bone loss on X-rays involves dentists visually analyzing intra-oral radiographs; this method is prone to errors [1, 13–15]. Detection of significant alveolar bone loss is one of the most common applications of DL models in dentistry; however, comparison among the models is challenging given the differences in types of intra-oral radiographs (e.g., bitewing, periapical), criteria for the ground truth, DL models used, and methods used to assess outcome [9, 12–14, 16, 17]. In this report, we detail the development and training of state-of-the-art DL algorithms that are proven to outperform previously studied neural networks, such as Visual Geometry Group (VGG) and ResNet neural networks [18, 19]. In contrast to some of the commercial applications, we leveraged training, validation, and test datasets that have been carefully curated by an oral radiologist.

There are several commercial programs available to dental providers incorporating AI for reading dental radiographs for caries and alveolar bone loss diagnosis, but very few studies have surveyed dental professionals about acceptability of specific applications, and explored how they intend to incorporate these advances into their practice [20]. Therefore, the objectives of this study were (1) to compare the accuracy and efficiency of the DL model for measurement of ACH compared to manual evaluations by providers, and (2) to explore whether an AI application for ACH measurement would be acceptable to dental providers and what aspects of the application they would find to be most helpful in their practice.

## Methods

### Development of artificial intelligence-deep learning model

**Fig. 1:**
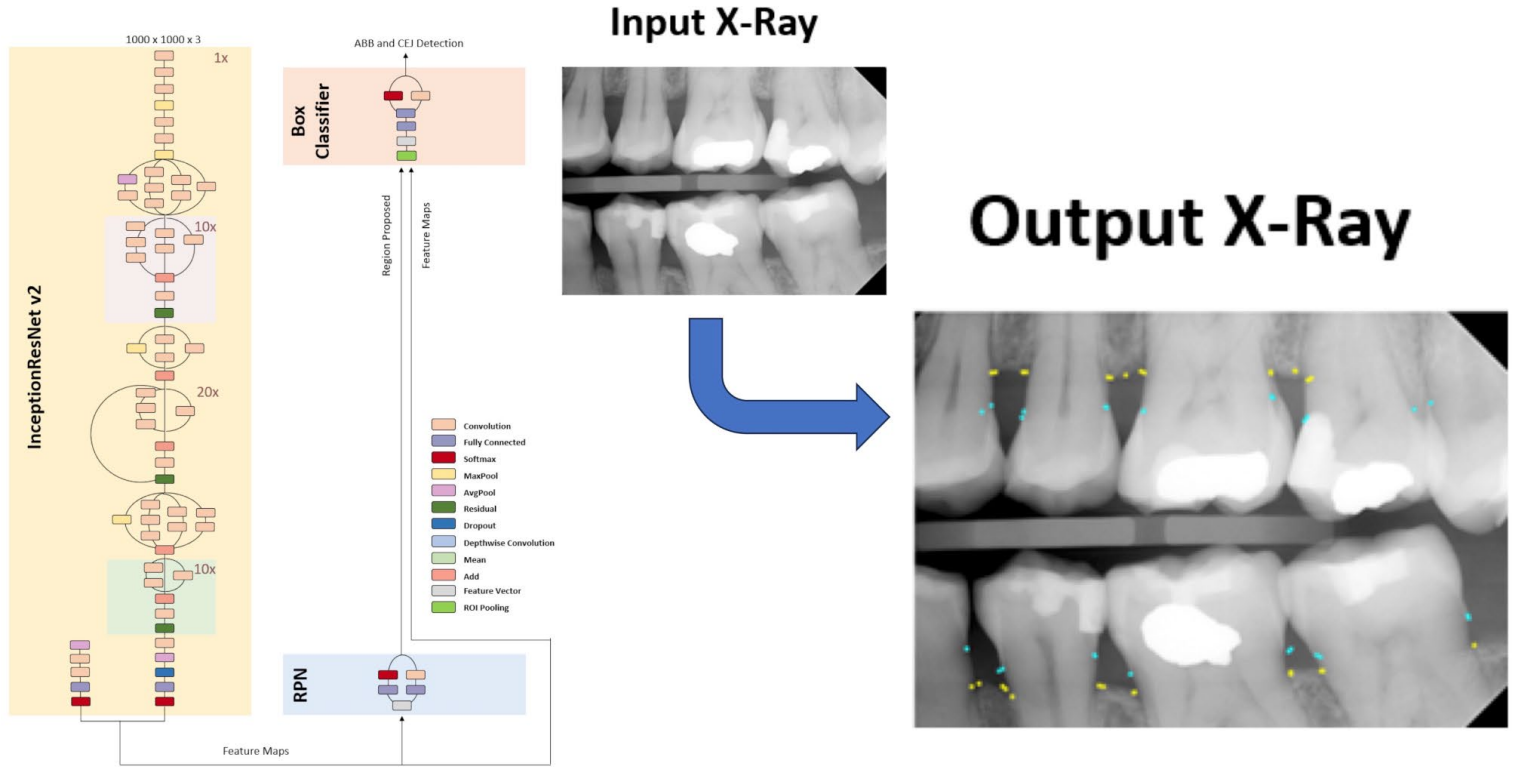
Neural Network Architecture for Calculating Alveolar Crestal Height (ACH)

A conglomerate of five deep neural networks was used to automate the measurement of ACH levels in bitewing radiographs. Initially, two object detection networks were employed to locate the coordinates of the alveolar bone crestal level (ABCL) and cemental-enamel junctions (CEJ) in the input radiographs. Subsequently, a semantic segmentation neural network was used to determine the pixel positions of ABCL, following which a best-fit line was drawn between the ABCL of the maxillary and mandibular arches to partition the upper and lower ABCL and CEJ. Polynomial curves were then fitted to the ABCL and CEJ points of both arches. Further, two semantic segmentation neural networks were utilized to identify the pixel locations of teeth in the radiographs. The tooth outlines from the previous tooth segmentation were then used to draw the ACH (i.e., CEJ– ABCL) if the outlines crossed both polynomial lines. Finally, the ACH levels were superimposed on the original input bitewing radiographs and provided as outputs. An example X-ray output of the conglomerate networks is shown in Fig. 1.

The neural network uses Faster-RCNN architecture with RPN and Box Classifier with Inception-ResNet-V2 backbone to detect alveolar bone crestal level (ABCL) and cemento-enamel junctions (CEJ) to measure ACH.

### Object detection deep neural networks

Two object detection neural networks were coded in MATLAB. For each of the two networks, an Inception-ResNet-v2 network was transformed into a Faster-R-CNN network by adding a region proposal network (RPN), regions of interest (ROI) max pooling layer, classification, and regression layers to support object detection [12, 14, 18, 21–23]. The key elements of Faster R-CNN are the RPN and the region-based convolutional neural network (CNN) (Fig. 1). The RPN generates a collection of potential object regions, also known as ROI, that are likely to contain an object. Subsequently, the region-based CNN analyzes each ROI to classify it into distinct object categories and optimize its bounding box coordinates. The fully convolutional RPN is fed an image as input, and it generates a list of rectangular object proposals, along with their corresponding objectness scores. By sweeping a sliding window over the image, the network produces a group of k-anchor boxes (bounding boxes of various sizes and aspect ratios that are predetermined). For each anchor box, the RPN computes the likelihood of it containing an object, along with the displacement required to align with the object’s ground-truth bounding box. These predictions are used to form a group of ROI which are then forwarded to the region-based CNN. The rectified linear unit (ReLu) at the 625th layer was used as a feature extraction layer, and an ROI pooling layer with an output size of [17 17] was inserted after this feature layer.

The object detection neural networks were trained, validated, and tested on 550 bitewing radiograph images that were annotated by a Board Certified Oral Radiologist with over 20 years of experience from Columbia University College of Dental Medicine. The oral radiologist evaluation of ACH calculations was repeated on 10 random selected X-rays and the intraclass correlation coefficient (ICC) for intra-rater reliability was 0.98, *P* < 0.001. All of the images were captured by a single x-ray unit which is calibrated annually for X-ray field size, reproducibility, kVp accuracy, radiographic timer accuracy, linearity, beam quality (half-value layer), and entrance skin exposure by radiation physicists from the Environmental Health and Safety, Radiation Safety Office at Columbia University. The 550 bitewing radiographs were randomly selected from a database of de-identified bitewing radiographs from 387 patients that were not edentulous with serial dental evaluations obtained between 12/1/2019 and 8/24/2020 in a previous study [24].

The images were randomly partitioned into training, validation, and testing sets in a 7:2:1 ratio for each of the two neural networks. The ABCLs and CEJs were enclosed by 60 × 60 pixel boxes, and the coordinates of these boxes were used in the training, validation, and testing sets. All images were resized to [869 1200 3] because the first object detection neural network layer was modified to accept this specific dimension. One neural network was trained for 20 epochs with mini-batch sizes of 2 and an initial learning rate of 1e-3. Another neural network was trained for 10 epochs with equal parameters. The object detection models were trained to predict optimal boxes centered on the ABCL and CEJ of teeth from an image, the centers of which were interpreted as the location of the ABCL and CEJ and subsequently noted by small circles with colors corresponding to their label in the output image. Due to the random partitioning of the training, validation, and testing sets, both networks were utilized for detecting ABCLs and CEJs as they demonstrated different levels of performance. Specifically, one of the networks achieved an average precision of 0.60 for all recalls in detecting ABCLs and an average precision of 0.65 for all recalls in detecting CEJs. On the other hand, the other network achieved an average precision of 0.60 for all recalls in detecting ABCLs and an average precision of 0.72 for all recalls in detecting CEJs. Both networks were employed to address the limitations of each other in detecting the ABCLs and CEJs in certain regions. The object detection models were trained on an Nvidia Titan X graphic processing unit (GPU).

### Semantic segmentation deep neural networks

Three semantic segmentation deep neural networks were coded in MATLAB. DeepLab v3 + CNNs for semantic image segmentation were created using Inception-ResNet-v2 as a base network [18, 25, 26]. The input layer was engineered to take input bitewing radiographs with a size of [1000 1000 3] for the network that segments ABCL, and [500 500 3] for the networks that segment teeth. One deep neural network was tasked with creating semantic masks of ABCL; thus, the network was modified to match the output label classes: ABCL and non-ABCL. Two deep neural networks were tasked with creating semantic masks for teeth; thus, the networks were modified to match the output label classes: teeth and non-teeth. The random partitioning of the training, validation, and testing sets resulted in the utilization of both networks for segmenting teeth, as they exhibited varying levels of performance. One of the networks achieved a global accuracy of 0.9567, while the other network achieved a global accuracy of 0.9562. Both networks were employed to address the limitations of each other in detecting the segmentation of teeth in certain regions [27, 28].

The DeepLab v3 + networks were designed using an encoder-decoder architecture, which involves encoding the input bitewing radiograph image into a lower-dimensional representation and then decoding it to generate the output segmentation mask [26]. This architecture was preferred for the semantic segmentation models because it can handle large bitewing radiograph sizes while still preserving fine-grained details. In addition to the encoder-decoder architecture, dilated convolutions were used to increase the receptive field of image of the network, enabling it to capture contextual information from a larger area in the input bitewing radiographs. Skip connections — which connect the output of the encoder to the decoder without downsampling — were implemented to help preserve high-resolution features in the overall output. Overall, the combination of these techniques and the Inception-ResNet-v2 base network resulted in a powerful semantic segmentation network capable of accurately segmenting bitewing radiographs.

To train, validate, and test the networks, a total of 550 bitewing radiographs were used. Label images corresponding to the bitewing radiographs were manually created with masks identifying ABCLs at pixel level for the semantic segmentation neural network that was tasked with segmenting ABCL. Label images corresponding to the bitewing radiographs were also manually created with masks identifying teeth at pixel level for the semantic segmentation neural networks that were tasked with segmenting teeth. DeepLab v3 + was trained using 60% of the bitewing radiographs from the dataset. The rest of the bitewing radiographs were split evenly into 20% each for validation and testing, respectively. To balance the classes for the training images, median frequency class weights were used by counting pixel labels, and the classes were weighed accordingly. Stochastic gradient descent with momentum was used for training. The neural network for ABCL segmentation was trained for 146 epochs with mini-batch sizes of 4 and an initial learning rate of 1e-3. The neural networks for teeth segmentation were trained for 150 epochs with mini-batch sizes of 4 and an initial learning rate of 1e-3. The segmentation models were trained using an Nvidia Titan RTX GPU.

### Computer application: deep learning results

In this study, two distinct semantic segmentation DL algorithms were utilized for specific tasks. The first algorithm aimed to segment teeth, achieving a global test set accuracy of 0.9567. The second algorithm focused on segmenting alveolar bone and attained a commendable global test set accuracy of 0.9281. For object detection, two separate algorithms were employed. The highest average precision of all recalls achieved by an algorithm, designed for detecting cemento-enamel junctions, was 0.72. The second algorithm, intended for detecting ABCLs, achieved the highest average precision of all recalls at 0.65.

### Survey of dental professionals to evaluate accuracy acceptability of AI application

A twenty-question survey was created to compare the accuracy and efficiency of manual X-ray examination versus the application and to assess the acceptability and usability of the application. To refine the survey and to assess reliability, we examined the consistency with which 5 dental providers differing by subspecialty and years of professional experience replied to the various items, prior to finalization. Responses were consistent throughout the measures obtained, arguing for stability across the items used. Evidence of content validity was provided by the lack of questions and comments in response to the instrument’s items, indicating that the instrument’s content fit well with the nature measurement of ACH being assessed. This survey was sent to all current and former members of Columbia Dental School’s community that includes orthodontists, general dentists, dental students, periodontists, and endodontists and consisted of 3880 email addresses. In the initial email we had 25 responses and on the second email we received an additional 39 responses, for a total of 64 responses, representing a 1.6% response rate. However 8 of the participants did not complete X-ray measurements. The survey was designed for this study and was not previously published elsewhere. The survey was designed to ask questions that gave a general overview of the level of expertise of the participant while also testing their knowledge and efficiency at identifying alveolar bone loss using ACH measurement through test bitewing radiographs. It also presented each participant with our computer program’s reading of the bitewing radiograph and asked them to judge its evaluation of the bitewing radiograph (Fig. 1). The accuracy results of the survey were then compared to the AI accuracy, with both being compared to the gold standard measures from an oral radiologist from Columbia School of Dental Medicine (SM).

To evaluate the expertise level of each participant, the survey was designed to inquire about the dentistry practice area of the participant, their professional setting, their duration of practice, and whether they use software to assist in the reading of X-rays in their practice. The survey then presented each participant with the same three bitewing radiographs and asked them to read and evaluate the severity of bone loss by measuring the ACH for both the mesial and distal side of each tooth, defining a measurement of < 5 mm as not severe and one of ≥ 5 mm as severe [29]. After each test bitewing radiograph, the participant was asked to list the number of minutes it took them to read the bitewing radiograph. The survey also introduced them to our AI computer algorithm that read the images itself, asking them to evaluate the accuracy of the program and any major differences between their readings and the computer’s.

This study was approved by the Columbia University Institutional Review Board (IRB), reference number AAAT2272.

### Statistical analysis

We conducted descriptive analyses comparing responses in surveys between groups of dental professionals. Continuous variables were described using means and standard deviations. Categorical variables were described with counts and percentages. In bivariate analyses, categorical variables were compared using the Wald chi-square test or Fisher exact test, and continuous variables were compared using the Mann-Whitney-Wilcoxon test or Kruskal-Wallis test as appropriate. All statistical analyses and data visualization were performed in SAS STAT software, version 13.2 (Cary, NC, USA).

## Results

In this study, we compared the measurements of ACH on all visible teeth in three different bitewing radiographs by 56 different dental professionals against the AI application’s performance. The evaluation focused on the application’s ability and dental professionals’ ability, respectively, to classify severe (ACH > 5 mm) vs. non-severe (ACH ≤ 5 mm) periodontal bone loss using 35 calculable ACHs present in three bitewing radiographs of the tooth.

**Fig. 2:**
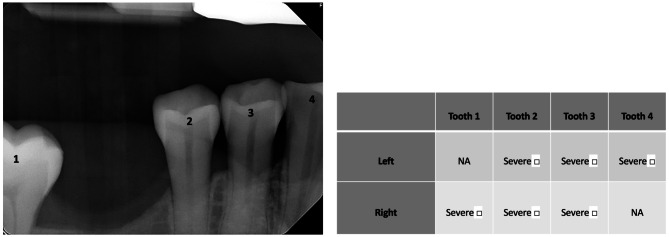
Sample question from survey: Participants were asked to evaluate the alveolar crestal height of each tooth and indicate whether there was severe bone loss (*≥* 5 mm ACH) by using check marks in the table below. Participants were also asked to indicate duration of time needed to perform the task.

The application achieved 94% accuracy in its classifications, in comparison to the 68% accuracy of the dental professionals (Fig. 2). In the first bitewing radiograph, there were 16 calculable ACHs. Dental professionals correctly classified 87% of teeth with severe periodontal disease (SD = 16%), while the AI application demonstrated perfect accuracy with 100% correct classifications. The mean estimated time to analyze the bitewing radiographs was 105.3 s (SD = 68.7) with a median of 60 s among dental professionals. In the second bitewing radiograph, there were 6 calculable ACHs. Dental professionals accurately identified only 35% of teeth with severe periodontal disease (SD = 29), whereas the AI application achieved an 83% accuracy rate. The mean estimated time to analyze the bitewing radiograph was 71.2 s (SD = 42.6) with a median of 60 s. In the third bitewing radiograph, there were 13 calculable ACHs. Dental professionals correctly classified 82% of teeth with severe periodontal disease (SD = 13), and the AI application achieved a 100% accuracy rate. The mean estimated time to analyze the bitewing radiograph was 80.6 s (SD = 52) with a median of 60 s. All images took less than 10 s for the AI application.

### Acceptability of AI software from dental professionals

**Fig. 3:**
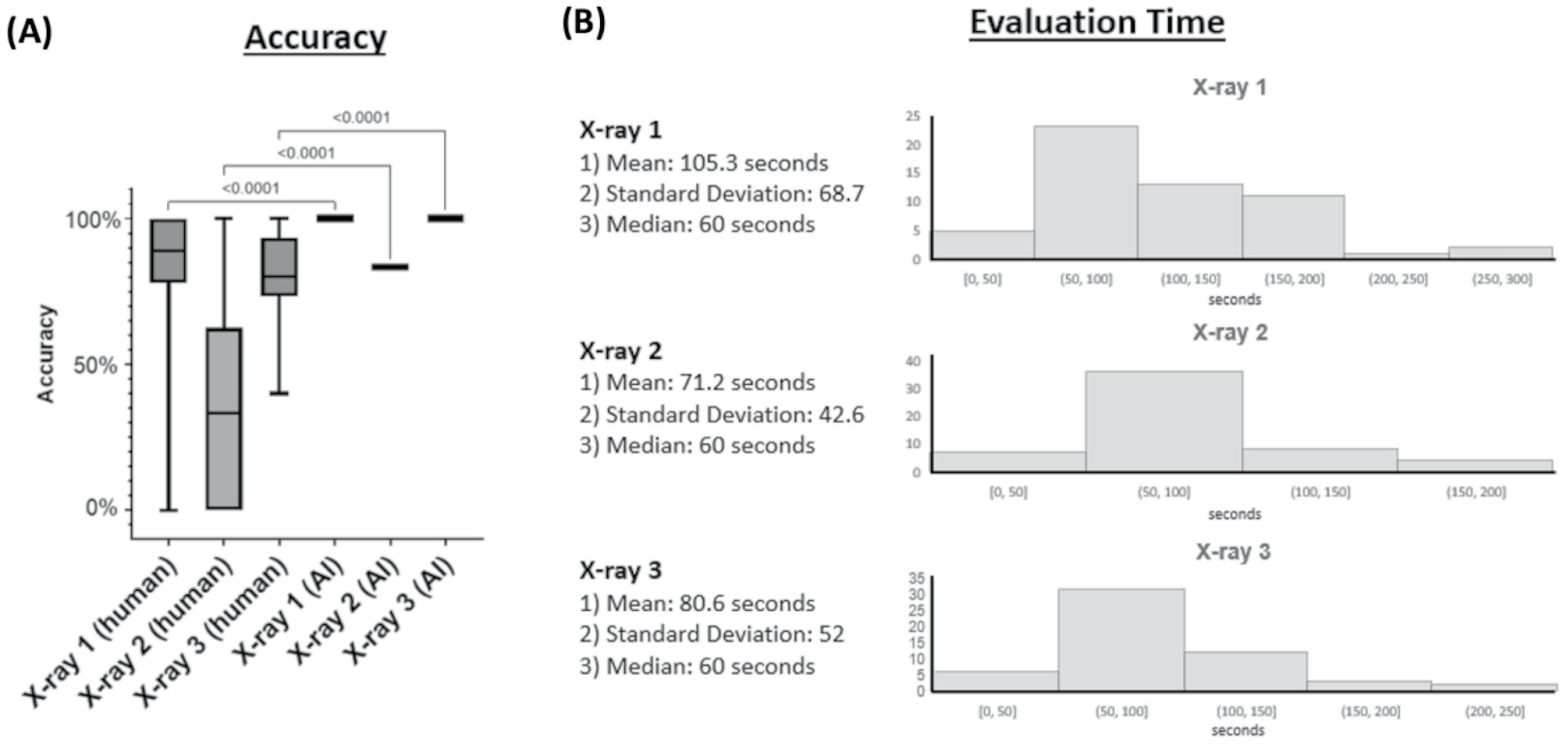
Comparison of accuracy and evaluation time between AI application and dental professionals (*n* = 56): (**A**) Accuracy: comparing range of responses from dental professionals and AI application. (**B**) Estimated time for X-ray evaluation by dental professionals: mean, standard deviation, median, and histograms.

**Fig. 4:**
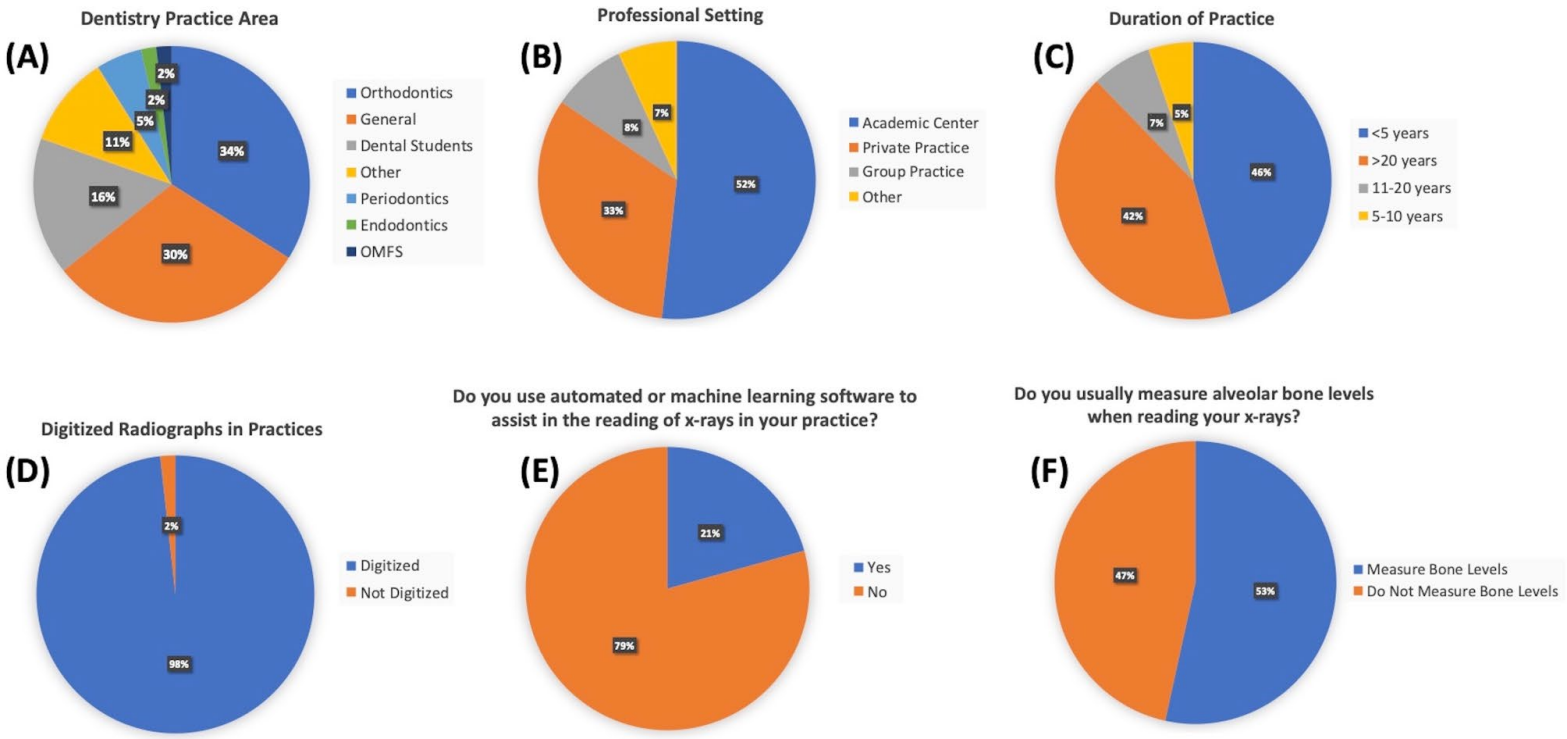
Survey Results. Columbia Dental School alumni were surveyed regarding their dentistry background and their methods for reading X-rays (*n* = 64). This background information included differences in (**A**) dentistry specialty, (**B**) setting of practice, (**C**) duration of practice, (**D**) whether radiographs are digitized in their practice, (**E**) whether or not machine learning is used in assisting practice, and (**F**) whether or not bone levels are measured when reading X-rays.

In total, the dental survey was completed by 64 participants, but the ACH measurement results were completed by 56/64 (87.5%) of participants (Figs. 3 and 4). Of the responses, 34% came from orthodontists, 30% from general dentists, and 16% from dental students. The remaining 20% of responses came from other dental professions including periodontists and endodontists. More than half the participants (52%) came from an academic setting, while 33% came from private practices, 8% came from group practices, and 7% came from other dentistry work settings. Only 21% of participants reported that they already used automated or AI-based software in their practice to assist in reading of bitewing radiographs. The majority, 57%, stated that they only approximate when measuring bone levels and only 9% stated that they measure with a ruler. The survey indicated that 84% of participants agreed or strongly agreed with the AI application measurement of ACH. Furthermore, 56% of participants agreed that AI would be helpful in their professional setting.

## Discussion

The DL model’s accuracy results, when compared to dental professionals, highlight its immense potential as a tool for precisely assessing severe versus non-severe ACHs and, thereby, periodontal disease-associated bone loss both in clinical practice and for research purposes. Similar to the majority of other studies, our model surpassed the measurement capabilities of dental professionals in this specific diagnostic task [30, 31]. Various medical fields have benefited from DL models being able to identify anatomical structures and detect pathological findings in radiographic images [1, 2, 4–12]. Some studies have attempted to measure the alveolar bone level by analyzing panoramic radiographs with DL models [1, 6, 7, 13, 16, 17, 32]. Panoramic images provide a quick overview of the dentition, but they suffer from considerable distortion and a lack of detail, making accurate and precise diagnosis of periodontitis and other oral diseases difficult [1, 13, 14, 21, 22, 28, 33]. The current standard practice of visually evaluating intra-oral radiographs is prone to inconsistency and errors [34, 35]. Advanced DL models on bitewing and/or periapical radiogrpahs can provide an objective and dependable method for periodontal diagnosis [36, 37]. Kim, et al. utilized multiple U-nets to gauge the alveolar bone level on bitewing radiographs [16]; however, comparison with state-of-the-art models is missing as there are many variants of U-Net that could have yielded higher accuracy [33].

Despite the significant advances in DL-based computer-aided diagnosis for oral imaging, its implementation has been limited [1–3]. This may be because there are only a few studies that have examined dental professionals’ acceptability and usability of these applications. In our study, we found that utilizing the DL model could significantly save time for dental professionals when calculating and charting ACHs from bite-wing radiographs which was similar to another study that used panoramic radiographs [38]. Similar to other studies we found that the majority of dental professionals (57%) stated that they only approximate when measuring bone levels, which is not ideal for longitudinal assessment for alveolar bone loss [39]. We also found that 56% participants agreed that AI would be helpful in their professional setting which is similar to other studies conducted in Croatia, where 71.0% of dentists surveyed believed AI technologies could enhance patient care, and in India, where 54% of dentist support AI in dental treatment planning [40]. Despite the enthusiasm, only 21% of our dental professionals reported that they already used automated or AI-based software in their practice to assist in reading of dental X-rays which is similar to what was reported in Croatia [41]. This presents a great opportunity for the widespread use of DL algorithms in dental practices.

Our team has developed and implemented an advanced DL model that not only employs semantic segmentation neural networks but also object detection networks to precisely identify the ABCLs and CEJs for periodontal disease detection. The model was trained and validated using datasets curated by an Oral Radiologist (SM) from Columbia University College of Dental Medicine to set a gold standard for ACH measurements. However, there are several important limitations to our model. The model has only been trained and validated on bitewing radiographs from a single brand of dental X-ray machines utilized in a single institution. We have not yet examined the program’s accuracy on radiographs taken with different equipment or by technicians at other institutions with varying levels of experience. Our survey of acceptability was exploratory in nature and the interpretation is limited by a low response rate, unequal representation of dental subspecialties and range of career experience. Future studies that are larger and more comprehensive are still needed to identify and address the barriers that our preventing dental professionals from using AI in their dental practice [42].

Overall, our study suggests that DL programs have the potential to accurately measure alveolar bone loss. We also demonstrate high acceptability among dental professionals. Given the clear potential benefits of increased accuracy and time saving for common clinical decisions, the availability and utilization of artificial intelligence applications in dentistry will undoubtedly increase.

## Electronic supplementary material

Below is the link to the electronic supplementary material.

Supplementary Material 1

## Data Availability

Additional data related to this paper may be requested from the authors.

## Abbreviations

DL: Deep learning
ABCLs: Alveolar bone crestal levels
CEJs: Cemento-enamel junctions
ACH: Alveolar crestal height
AI: Artificial intelligence
VGG: Visual Geometry Group
RPN: Region proposal network
CNN: Convolutional neural network
ReLu: Rectified linear unit
GPU: Graphic processing unit
IRB: Institutional Review Board
ROI: Regions of Interest
