# Supplementary material for: Evaluation by dental professionals of an artificial intelligence-based application to measure alveolar bone loss
Source: BMC Oral Health. 2025 Mar 1;25:329. doi: 10.1186/s12903-025-05677-0 (PMC11872301; doi:10.1186/s12903-025-05677-0)
Supplement: Supplementary file 1 — Supplementary Material 1 [file 12903_2025_5677_MOESM1_ESM.docx]

**Provider Survey**

1. In what area of dentistry do you practice?
   1. General
   2. Orthodontics
   3. Periodontics
   4. Endodontics
   5. OMFS
   6. Dental Resident
   7. Dental Student
   8. Other
2. What kind of setting do you work in?
   1. Private practice
   2. Group practice
   3. Academic center
   4. Other
3. How many years are you out of dental school
   1. <5 years
   2. 5-10 years
   3. 11-20 years
   4. >20 years
4. Do you use digitalized radiographs in your practice?
   1. Yes
   2. No
5. Do you use automated or machine learning software to assist in the reading of x-rays in your practice?
   1. Yes
   2. No
6. Do you usually measure alveolar bone levels when reading your x-rays?
   1. Yes
   2. No
7. If yes how do you measure the bone levels
8. Approximate
9. Measure with ruler
10. Other ----

For questions 8-12, the teeth are numbered in the direction from left-to-right and top-to-bottom. Please use the following definitions for the severity of bone loss by measurement of alveolar crestal height (ACH; distance from cemento-enamel junction to alveolar bone crestal height) on either the mesial and/or distal side of each tooth.

|  | Not Severe | Severe |
| --- | --- | --- |
| Severity | <5 mm | ≥5 mm |

1. Please read the x-ray below and evaluate ACH for each tooth. Indicate severe bone loss (>5 mm) by ACH using check marks in the table below. Please time how long it takes for you to analyze the x-ray.


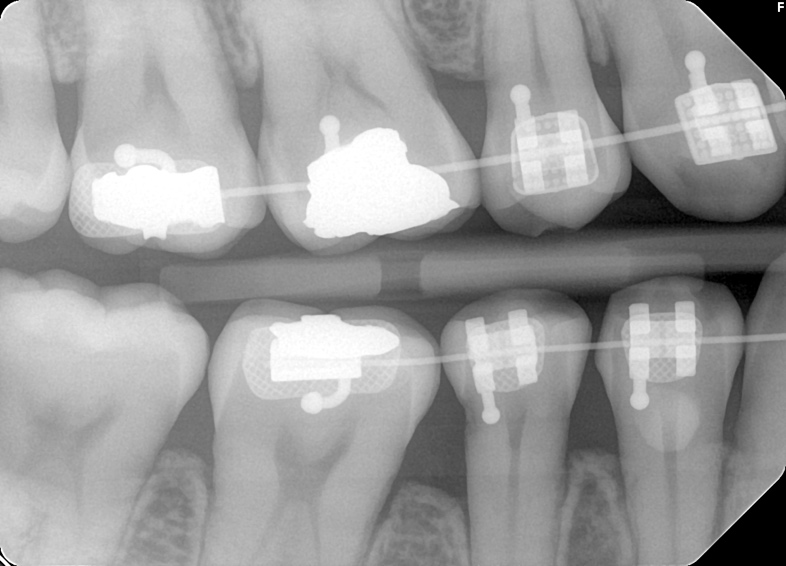

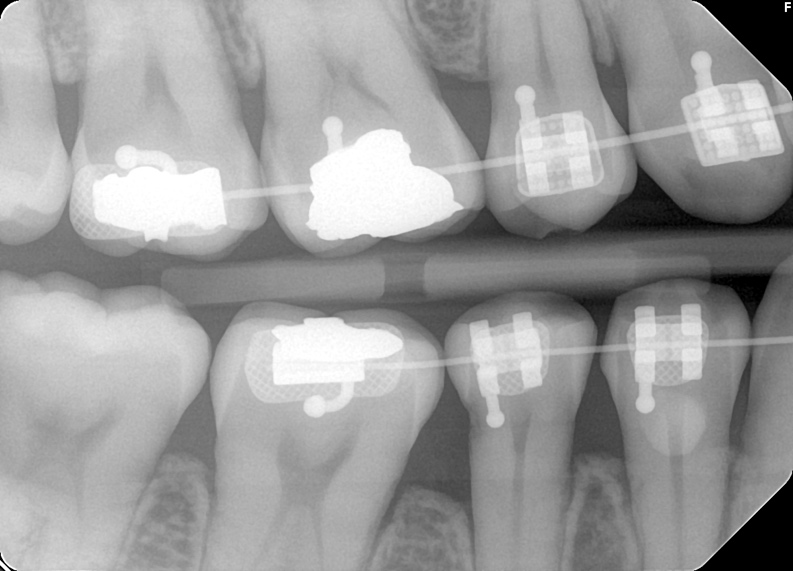


**10**

**9**

**8**

**7**

**6**

**5**

**4**

**3**

**2**

**1**

|  | Tooth 1 | Tooth 2 | Tooth 3 | Tooth 4 | Tooth 5 | Tooth 6 | Tooth 7 | Tooth 8 | Tooth 9 | Tooth 10 |
| --- | --- | --- | --- | --- | --- | --- | --- | --- | --- | --- |
| Left | NA | Severe □ | Severe □ | Severe □ | Severe □ | Severe □ | Severe □ | Severe □ | Severe □ | Severe □ |
| Right | Severe □ | Severe □ | Severe □ | Severe □ | Severe □ | Severe □ | Severe □ | Severe □ | Severe □ | NA |

- 1. How long did it take to read the x-ray (minutes)? ________

1. Please read the x-ray below and evaluate ACH for each tooth. I Indicate severe bone loss (>5 mm) by ACH using check marks in the table below. Please time how long it takes for you to analyze the x-ray.


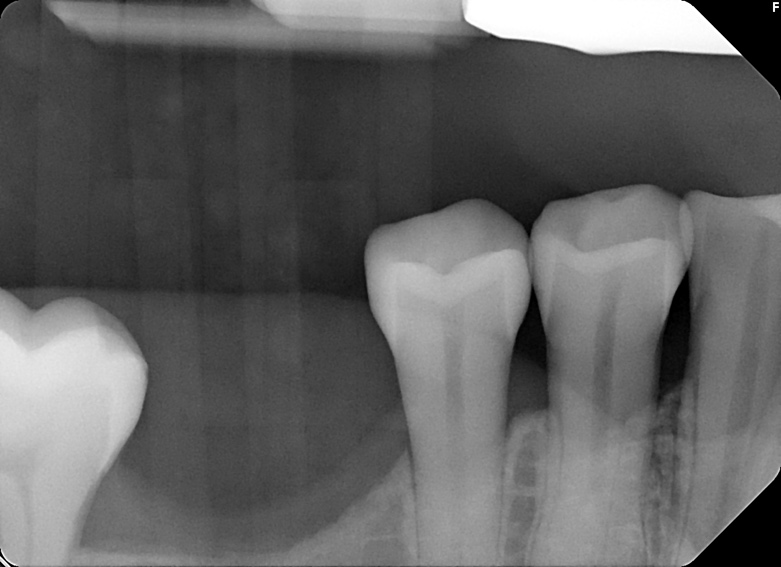

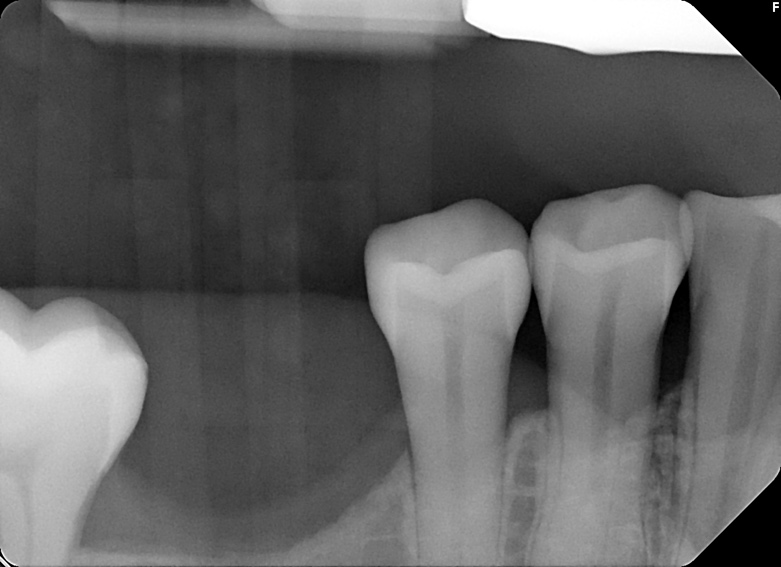


**2**

**4**

**1**

**3**

**7**

|  | Tooth 1 | Tooth 2 | Tooth 3 | Tooth 4 |
| --- | --- | --- | --- | --- |
| Left | NA | Severe □ | Severe □ | Severe □ |
| Right | Severe □ | Severe □ | Severe □ | NA |

- 1. How long did it take to read the x-ray (minutes)? ________

1. Please read the x-ray below and evaluate ACH for each tooth. Indicate severe bone loss (>5 mm) by ACH using check marks in the table below. Please time how long it takes for you to analyze the x-ray.


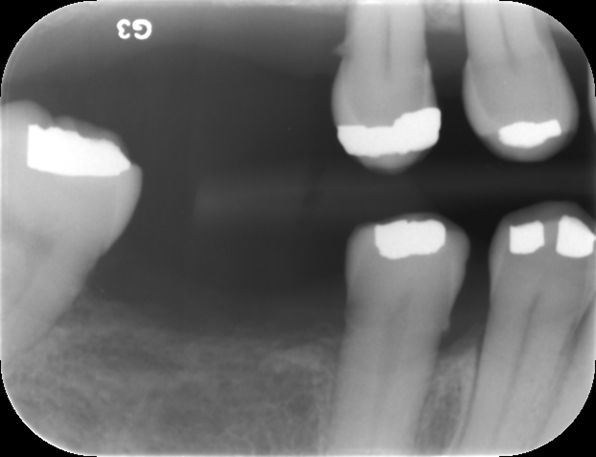

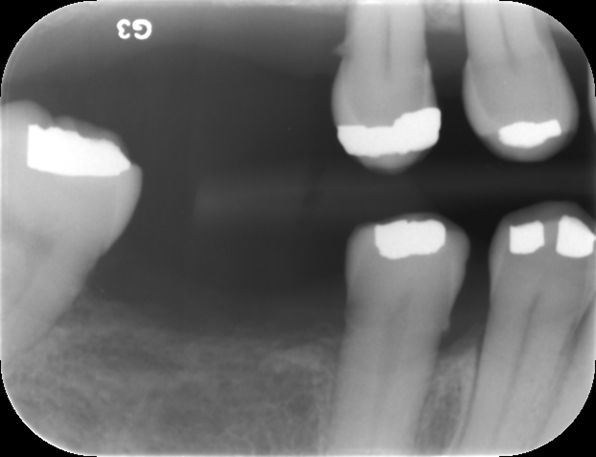


**2**

**1**

**4**

**3**

|  | Tooth 1 | Tooth 2 | Tooth 3 | Tooth 4 |
| --- | --- | --- | --- | --- |
| Left | Severe □ | Severe □ | Severe □ | Severe □ |
| Right | Severe □ | Severe □ | Severe □ | NA |

- 1. How long did it take to read the x-ray (minutes)? ________

1. Please read the x-ray below and evaluate ACH for each tooth. This x-ray was taken at a two different time points for the same patient seen in the previous question (Question #12). Please indicate whether the change in ACH in #10 from #11 was greater than 1 mm. Please time how long it takes for you to analyze the x-ray.


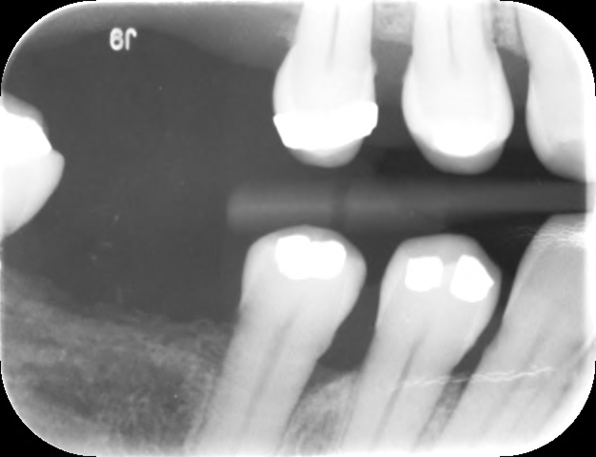

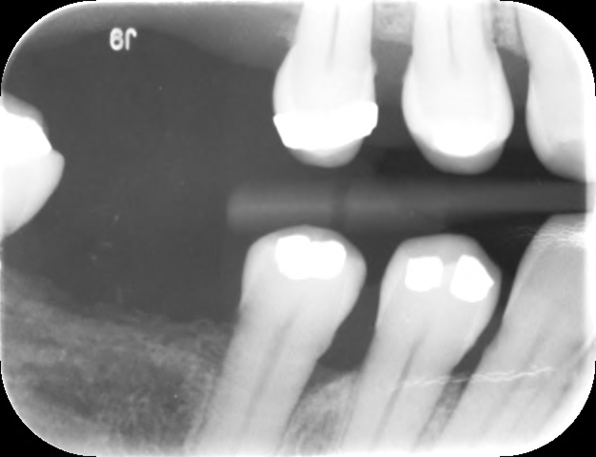


**4**

**3**

**2**

**1**

|  | Tooth 1 | Tooth 2 | Tooth 3 | Tooth 4 |
| --- | --- | --- | --- | --- |
| Left | △>1mm □ | △>1mm □ | △>1mm □ | △>1mm □ |
| Right | △>1mm □ | △>1mm □ | △>1mm □ | △>1mm □ |

- 1. How long did it take to read the x-ray (minutes)? ________

1. The alveolar crestal heights for the xrays in previous questions are shown below. To see how the artificial intelligence-based application calculates the ACH, please watch the attached 3-minute video.


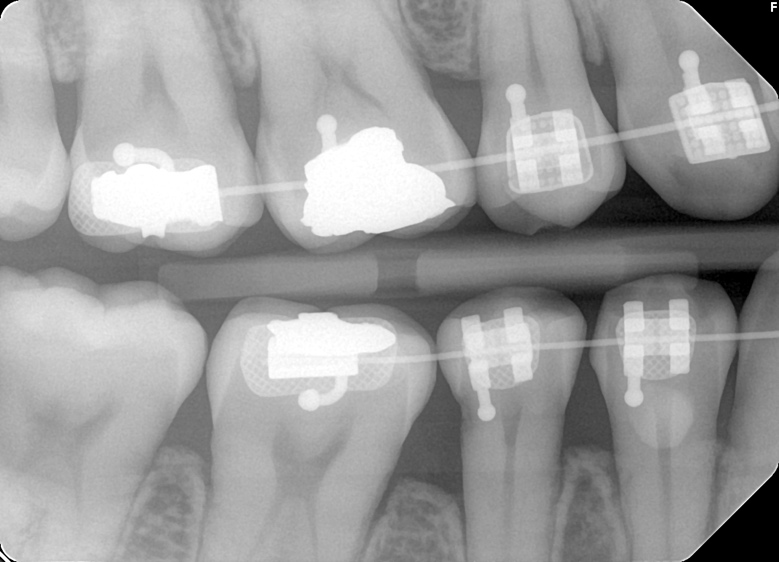

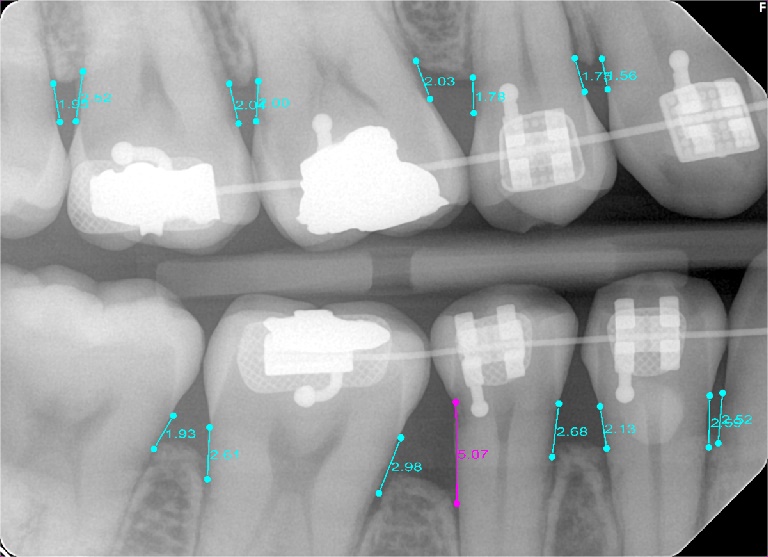


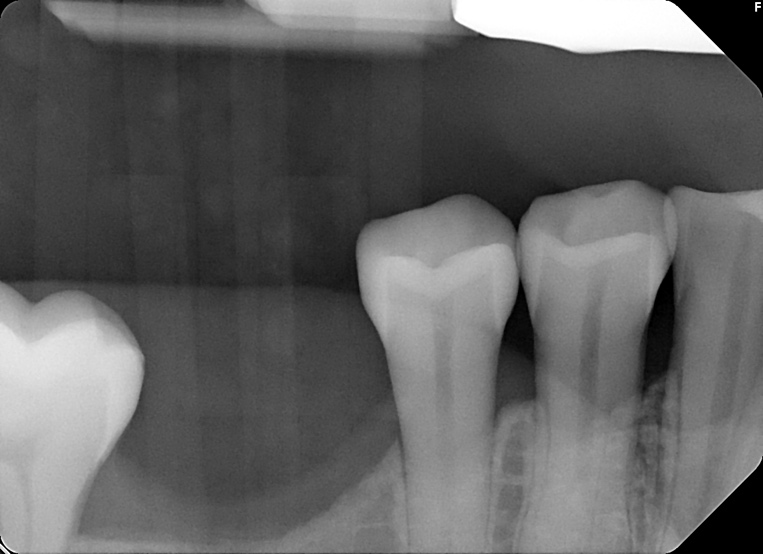

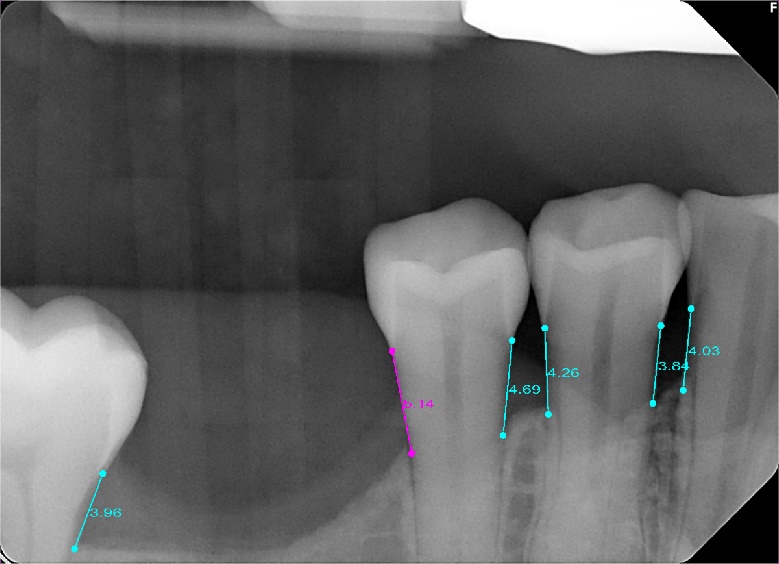


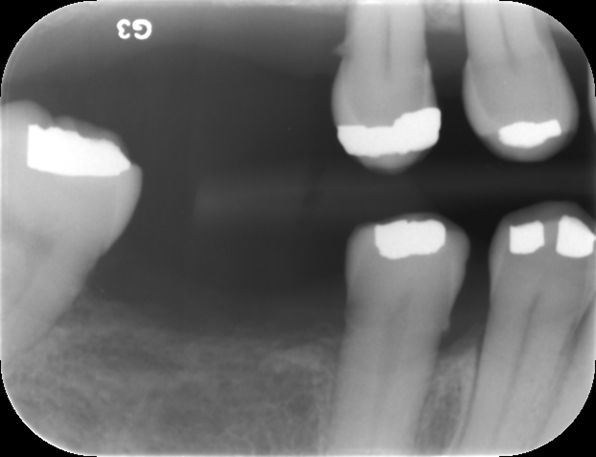

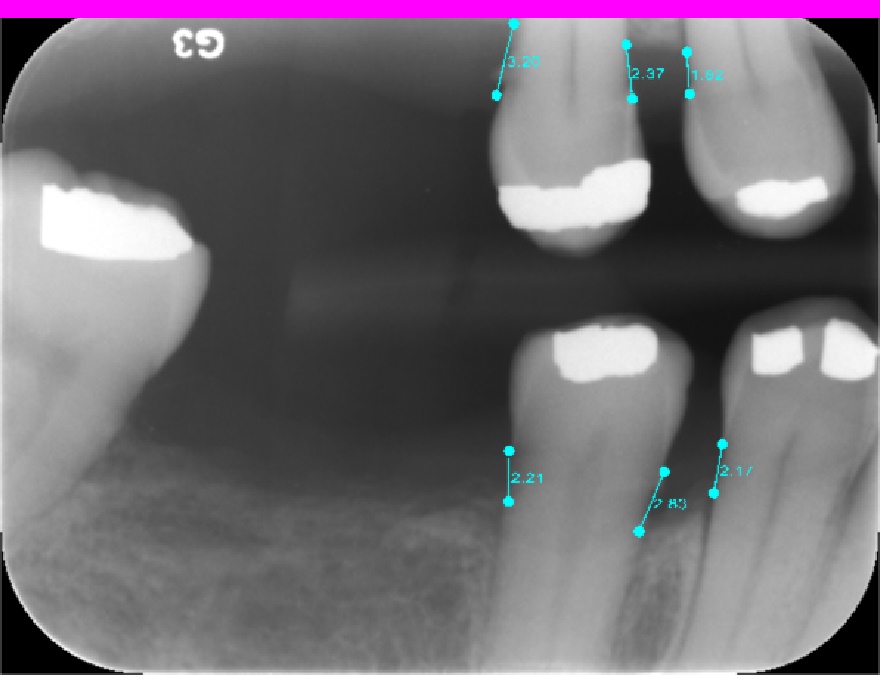


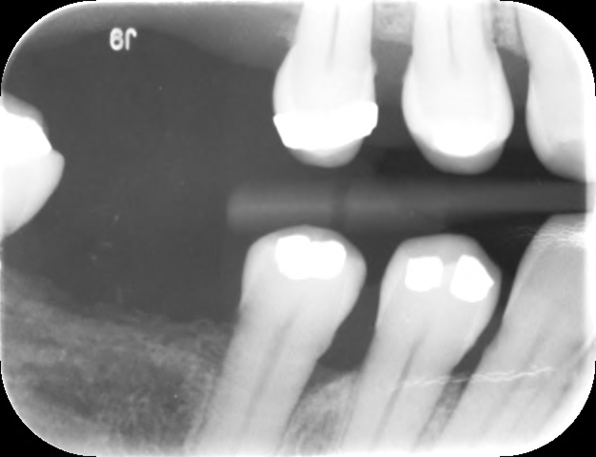

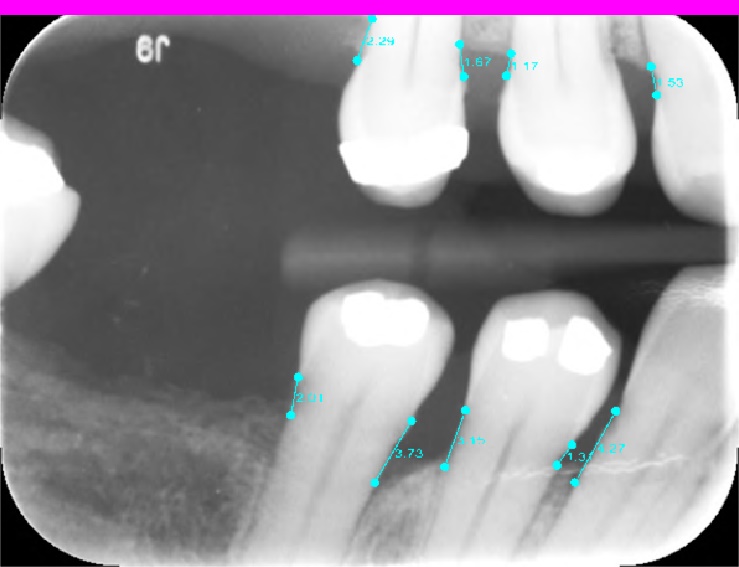


Were there major differences between your reading and the computer program reading?

- 1. Yes
  2. No

1. Do you believe that the computer program’s reading of alveolar crestal height is accurate?

a) Strongly Agree b) Agree c) Neutral d) Disagree e) Strongly Disagree

1. Do you think that the computer program would be helpful in your practice?

a) Strongly Agree b) Agree c) Neutral d) Disagree e) Strongly Disagree

1. In what ways do you think the computer program would benefit your practice? (Circle all that apply)
   1. Provides a faster read of the x-ray than a dentist
   2. Provides a more comprehensive read of all teeth in the x-ray
   3. The report provides objective measures that can be followed over time
   4. The report will help to justify the diagnosis of periodontal disease.
   5. Other: __________________________
2. Did you find the software easy to navigate and use in the video?

a) Strongly Agree b) Agree c) Neutral d) Disagree e) Strongly Disagree

1. What other information would you be interested in obtaining via automated software? (Circle all that apply)
   1. Caries detection
   2. Automatic charting
   3. Other: ________________________
2. Would you like the artificial intelligence-based application to automatically analyze the alveolar crestal heights every time a bitewing x-rays is taken at your practice?
   1. Yes
   2. No
3. We would like your opinion about how this computer program might benefit your specific specialty (i.e., orthodontist, etc.) either in its present form or with specific refinements. Please feel free to comment: ______________________
4. We would like your opinion about how to make this computer program more useful to dentists and easy to use. Please feel free to comment: ______________________
